# Neurodynamic profiles in the alpha band distinguish different forms of meditation

**DOI:** 10.1093/nc/niag036

**Published:** 2026-07-07

**Authors:** Bianca Ventura, Andrea Buccellato, Saketh Malipeddi, Rahul Venugopal, Ravindra P Nagendra, Bindu M Kutty, Georg Northoff

**Affiliations:** School of Psychology, University of Ottawa, 136 Jean-Jacques Lussier, Ottawa, ON K1N 6N5, Canada; The Royal’s Institute of Mental Health Research & University of Ottawa, Brain and Mind Research Institute, Centre for Neural Dynamics, Faculty of Medicine, University of Ottawa, 145 Carling Avenue, Rm. 6435, Ottawa, ON K1Z 7K4, Canada; The Royal’s Institute of Mental Health Research & University of Ottawa, Brain and Mind Research Institute, Centre for Neural Dynamics, Faculty of Medicine, University of Ottawa, 145 Carling Avenue, Rm. 6435, Ottawa, ON K1Z 7K4, Canada; Centre for Consciousness Studies, Department of Neurophysiology, National Institute of Mental Health and Neuro Sciences, Hosur Road, Bengaluru, 560029, Karnataka, India; Centre for Consciousness Studies, Department of Neurophysiology, National Institute of Mental Health and Neuro Sciences, Hosur Road, Bengaluru, 560029, Karnataka, India; Centre for Consciousness Studies, Department of Neurophysiology, National Institute of Mental Health and Neuro Sciences, Hosur Road, Bengaluru, 560029, Karnataka, India; Centre for Consciousness Studies, Department of Neurophysiology, National Institute of Mental Health and Neuro Sciences, Hosur Road, Bengaluru, 560029, Karnataka, India; The Royal’s Institute of Mental Health Research & University of Ottawa, Brain and Mind Research Institute, Centre for Neural Dynamics, Faculty of Medicine, University of Ottawa, 145 Carling Avenue, Rm. 6435, Ottawa, ON K1Z 7K4, Canada

**Keywords:** meditation techniques, Peak Frequency Sliding, Power Sliding, alpha oscillations, temporal dynamics

## Abstract

Different forms of meditation, like Vipassana and Shoonya, require distinct attentional features. For instance, Vipassana, which involves systematically scanning different body parts, may demand a sustained yet dynamic focus with a temporally stable and regular attentional emphasis. In contrast, Shoonya meditation has been described as involving a non-directed or defocused attentional stance, likely with a temporally variable and irregular attentional flow. We used electroencephalography (EEG) to examine whether these differences could correspond to distinct neurodynamic patterns in the alpha band. We measured two time-resolved dynamic measures including phase-based Peak Frequency Sliding (PFS), indexing fine-grained millisecond temporal resolution, and Power Sliding (PS), indexing oscillatory amplitude. Beyond mean values, we additionally quantified the temporal dynamics of PFS and PS using coefficient of variation (CV) as an index of variability, and permutation entropy (PE) as a measure of temporal irregularity. While mean differences between the two practices were observed only for PS (and not PFS), their dynamic variants more robustly distinguished the two practices. Specifically, Vipassana exhibited lower CV and PE in both PFS and PS compared to Shoonya. These findings indicate that the two practices differ more strongly in the temporal-dynamic organization of alpha-band activity than in their static averaged measures. More broadly, they support the view that dynamic features such as variability and temporal irregularity may be shared by both brain and experience, as their “common currency,” during different contemplative practices.

## Introduction

### Meditation techniques—the attentional dynamics continuum

Meditation practices are broadly conceived as techniques to foster awareness and equanimity for reducing suffering and cultivating a more harmonious relationship with oneself and the environment (Trautwein et al. 2014, Adventure-Heart and Proeve 2017, Ray et al. 2021). While this is a common goal among meditation techniques (Osho 2012, Reddy and Roy 2019, Cooper et al. 2022), the methods of inducing meditative states vary considerably, reflecting distinct philosophical and practical frameworks (West 1987, Koshikawa and Ichii 1996, Lutz et al. 2007, 2008, Lehmann et al. 2012, Awasthi 2013, Fox et al. 2016, Fucci et al. 2018, Fujino et al. 2018, Raffone et al. 2019, Woods et al. 2020, 2024). In particular, the induction methods differ in the type and quantity of their objects of attention (Valentine and Sweet 1999, Jha et al. 2007, Manna et al. 2010, Raffone and Srinivasan 2010, Amihai and Kozhevnikov 2015, Tsai and Chou 2016, Braboszcz et al. 2017, Brandmeyer et al. 2019, Prakash 2021, Kozhevnikov et al. 2022) and key temporal features, that is, attentional dynamics, including aperture, intensity of focus, clarity, and stability on the meditation object, and structure of the experiential flow (Lutz et al. 2008, Lippelt et al. 2014, Ventura et al. 2024).

For instance, body scan meditation, like Vipassana (see below), involves a narrow and intense focus that shifts systematically across different body regions in a temporally regular way (Chiesa 2010, Delgado-Pastor et al. 2013, Nash et al. 2013, Braboszcz et al. 2017). Focused attention meditation likewise requires a narrow and intense focus, but here it is maintained on a single object, thereby sustaining a temporally stable attentional stance with reduced susceptibility to distraction from competing stimuli (Manna et al. 2010, Fox et al. 2016, Braboszcz et al. 2017). In contrast, open monitoring meditation involves a broad and flexible attentional stance, allowing attention to move freely and more irregularly over time across bodily sensations, thoughts, emotions, and external stimuli, without fixation (Manna et al. 2010, Fox et al. 2016). Finally, in no-content meditation, such as Shoonya (see below), attentional selection is further removed, maintaining an open, objectless awareness in which experiences arise and pass with high temporal variability and irregularity, without deliberate monitoring or engagement, that is, a state of “conscious non-doing” (Nash et al. 2013, Pagnoni 2019, Laukkonen and Slagter 2021).

Previous research has shown that these distinct attentional styles correspond to different neuronal features, including band-power changes and large-scale network activity (Lomas et al. 2015, Fox et al. 2016, Braboszcz et al. 2017, Vivot et al. 2020). However, this body of work has predominantly characterized meditative states in a static manner, typically by averaging frequency-band power over time. As such, it largely overlooks the moment-to-moment temporal dynamics of neural oscillatory activity that may underlie key temporal features of attentional organization, such as variability and regularity, required by different meditation forms. To address this gap, the present study examined the neural dynamics underlying attentional-temporal differences across meditation practices. Specifically, we focused on two techniques that may represent opposite poles along a continuum of attentional styles: one characterized by a detailed, vivid, and stable attentional focus with low variability and high temporal regularity, and the other by a diffuse, unstructured, and contentless awareness marked by high variability and temporal irregularity. The former corresponds to Vipassana meditation, specifically the body scan practice rooted in the tradition of Sayagyi U Ba Khin and taught by S.N. Goenka (Hart 1987, Goenka 1995). The latter corresponds to Shoonya meditation, a no-content practice derived from yogic traditions and widely disseminated through Sadhguru’s Isha Yoga programs (Sadhguru 2016).

### Vipassana and Shoonya meditation—different dynamics in the temporal resolution and intensity of their attentional focus

Based on traditional descriptions of Vipassana meditation, attention is typically described as being directed in a highly focused and systematic manner across the body, moving sequentially from the top of the head downward or in the reverse direction, covering each body region in an ordered and temporally regular progression (Goenka 1995, Fox et al. 2016, Braboszcz et al. 2017, Kakumanu et al. 2018). The meditator is instructed to direct their attention to physical sensations arising from each scanned body area (such as tingling, pressure, warmth, or subtle vibrations), observing these experiences as they emerge and fade with high experiential intensity, while maintaining a neutral and equanimous awareness towards all of them (Goenka 1995). Such systematic observation has been described as gradually weakening habitual patterns of physical, emotional, and mental reactivity: instead of reacting to sensations with craving or aversion, meditators cultivate a deeper understanding of their transient nature, embodying the experiential realization of impermanence (Hart 1987).

Conversely, Shoonya meditation has been described as a practice of “conscious non-doing” that involves cultivating a state of effortless awareness and de-identification. The practice is said to aim at a state of emptiness or “Shoonya,” which translates to no-thingness (signifying a non-physical dimension) (Malipeddi et al. 2024). Unlike techniques that rely on structured observation or concentration, Shoonya is described as encouraging “conscious non-doing,” i.e. a deliberate disengagement from thoughts, emotions, and sensory inputs (Nash et al. 2013, Braboszcz et al. 2017, Pagnoni 2019, Vivot et al. 2020, Laukkonen and Slagter 2021, Malipeddi et al. 2024). Practitioners are typically guided to sit in a relaxed, yet alert posture and consciously let go of any effort to control or direct their mental focus: the emphasis is on allowing thoughts, sensations, and experiences to arise and dissipate naturally, without engaging the sense of self through identification with or reaction to them. The process requires an acute awareness of the present moment, coupled with letting go of the habitual urge to categorize, analyze, or grasp at experiences. Unlike body-centric or breath-centric practices, Shoonya does not tether attention to a specific object of focus; instead, it creates an expansive and unstructured field of awareness. Informed by traditional descriptions and prior phenomenological reports, and in the absence of empirically validated phenomenological measures in the present dataset, we operationally characterize Shoonya as involving comparatively lower attentional intensity and greater temporal variability and irregularity in the experiential flow than Vipassana; these are our working assumptions guiding our neural hypotheses (Sadhguru 2016, Malipeddi et al. 2024).

These two practices thus represent contrasting attentional-temporal profiles along multiple dimensions with high versus low variability and high versus low irregularity, and were selected precisely for this reason. It should be noted, however, that they also differ along dimensions beyond attentional structure, including posture, effort, breathing patterns, and propensity for drowsiness (Cahn and Polich 2006, Lutz et al. 2008), all of which could in principle influence oscillatory neural dynamics. The present study focuses specifically on the neurodynamics underlying their different attentional-temporal styles as the primary dimensions of interest, while potential confounds arising from these additional differences are addressed through the inclusion of relevant covariates in the statistical models (see Materials and methods and Supplementary Material).

### Peak Frequency Sliding and Power Sliding: different dynamics in the temporal resolution and intensity of neural activity

Meditative states have been consistently associated with modulations in alpha oscillations (7–13 Hz; Fell et al. 2010, Lomas et al. 2015, Kakumanu et al. 2018, Lee et al. 2018). Alpha oscillations primarily relate to sensory input processing, gating, and external cognition (Klimesch 2012, Wolff et al. 2019a, 2022, Golesorkhi et al. 2021, Hua et al. 2022, Buccellato et al. 2023). To examine the different attentional styles fostered by Vipassana and Shoonya meditation, we analyzed oscillatory alpha dynamics using Peak Frequency Sliding (PFS) and Power Sliding (PS), two measures of input processing as defined by Cohen (2014), allowing us to investigate the moment-to-moment neural dynamics of the alpha band in both its phase cycles (PFS) and amplitude (PS).

PFS, derived from the first derivative of the instantaneous phase-angle time series obtained through the Hilbert transform, is a phase-based measure that reflects the temporal “speed” of neural oscillations, providing insights into the underlying phase cycle duration. Cohen (2014) demonstrated that alpha PFS correlated with stimulus strength by encoding the input magnitude in neural ensembles; slower alpha oscillations enhanced neural sensitivity to weaker inputs, while faster alpha oscillations promoted more temporally precise responses. Research further showed that faster alpha PFS predicts more fine-grained temporal resolution in perception, supporting faster stimulus segregation and perceptual precision (Samaha and Postle 2015, Wutz et al. 2018, Drewes et al. 2022). Additionally, alpha PFS modulates perceptual ambiguity (Shen et al. 2019), decreases during sustained tasks (Benwell et al. 2019), tracks on-task thought dynamics (Hua et al. 2022), and exhibits scale-free fluctuations influenced by cognitive and sensory states (Nakao et al. 2019, Jia et al. 2023). Collectively, these findings suggest that faster alpha PFS may be associated with increased temporal resolution of neural processing, which, in turn, might support fine-grained perceptual discrimination with higher attentional-temporal precision.

Alpha PS captures the instantaneous amplitude of the signal, and is calculated by extracting the modulus (i.e. the absolute value) of the Hilbert transform. Alpha PS is inversely related with cortical excitability and associated with functional inhibition of task-irrelevant cortical regions (Romei et al. 2008, Haegens et al. 2011, Lange et al. 2013, Benwell et al. 2019). It seems to play a key role in attentional control by filtering out irrelevant information through increased alpha power, effectively regulating the flow of relevant and irrelevant inputs (Klimesch et al. 2007, Jensen and Mazaheri 2010, Fries 2015, Van Diepen et al. 2019). In addition, previous studies have shown that the amplitude of oscillatory activity within a given frequency band reflects the degree of synchrony among the underlying neural current sources (Pfurtscheller and Lopes da Silva 1999, Buzsáki 2006, Nunez and Srinivasan 2006, Srinivasan et al. 2007). This synchrony arises from the coordinated activity of neuronal populations oscillating at the same frequency, facilitating efficient neural communication. Importantly, alpha power is also sensitive to vigilance and arousal fluctuations (Klimesch 1999, Benwell et al. 2019), indicating that it reflects not only selective attentional modulation but also the overall stability of the ongoing brain state. Rather than viewing these interpretations as mutually exclusive, they may converge in contexts where sustained attentional engagement and intensity is supported by a globally aroused neural state.

In meditation research, increased alpha power has been associated with deeper meditation experiences and heightened concentration (Katyal and Goldin 2021; Lomas et al. 2015). Within this framework, higher alpha PS may reflect a condition in which distracting inputs are suppressed, neural populations oscillate more coherently, and the global state remains stable and regular, together enabling a sustained and intensified engagement with the meditation object. In this sense, alpha PS may index the intensity and stability of the neural signal supporting attentional focus, which could manifest phenomenologically as increased experiential vividness. Together, PFS and PS may capture complementary dimensions of alpha oscillatory dynamics, that is, their temporal resolution and intensity, respectively. These dimensions may differentially support the structured, fine-grained attentional scanning characteristic of Vipassana and the more diffuse, open, and potentially lower-intensity awareness cultivated in Shoonya meditation.

**Figure 1:**
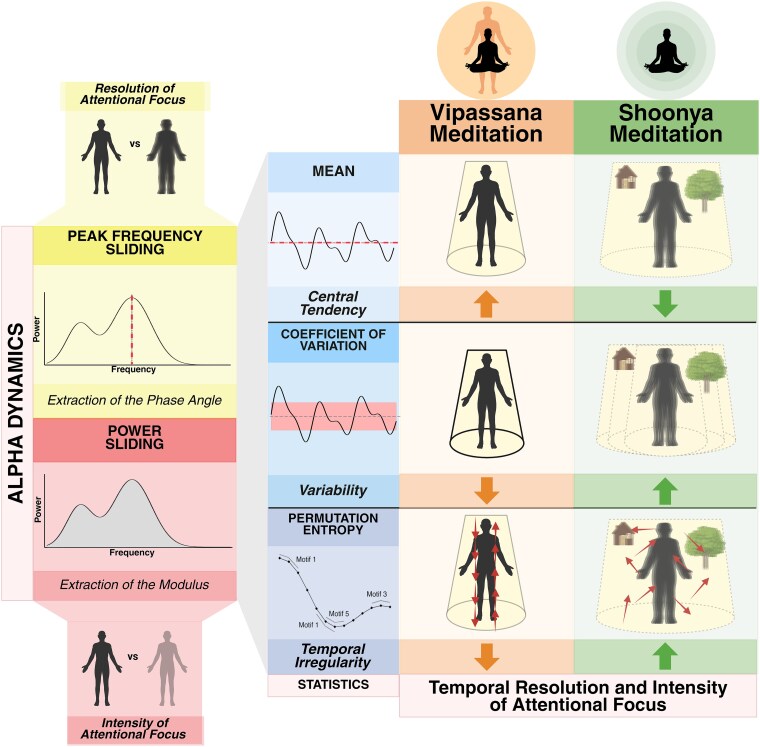
Conceptual framework illustrating the neural measures used to analyze the dynamics of meditative states in Vipassana and Shoonya practices, characterized by different attentional dynamics. Alpha oscillations are assessed through Peak Frequency Sliding (PFS) and Power Sliding (PS), possibly indicating the resolution and the intensity of attentional focus, respectively. Mean, coefficient of variation (CV), and permutation entropy (PE) are applied to PFS and PS time series, in order to characterize their central tendency, variability, and temporal irregularity. This approach allowed for a comprehensive investigation of alpha dynamics and their relationship to the phenomenological characteristics of the two meditation practices. Created in BioRender. Ventura, B. (2026). https://BioRender.com/qyzkakj.

The goal of the present study was to examine alpha PFS and PS as candidate neural indices of temporal resolution and intensity in Vipassana and Shoonya meditation, in line with their distinct attentional dynamics. Beyond characterizing these measures by their mean values, we were particularly interested in how they evolve dynamically over the course of the meditation session. Using PFS and PS approach to derive instantaneous estimates of alpha band (Cohen 2014), we investigated attentional-temporal differences between the two meditation practices at the neurodynamic level, focusing on variability and temporal regularity over time. To this end, we supplemented the standard mean with two additional dynamic indices applied to the PFS and PS time series: the coefficient of variation (CV) and permutation entropy (PE). The mean represents the central tendency averaged across its fluctuations over time in PFS and PS, while their CV quantifies their variability/consistency from time point to time point, offering insight into the stability of PFS and PS over time (Wolff et al. 2019a). Finally, PE measures the degree of temporal structure of PFS and PS, providing a metric for testing the diversity of ordinal patterns in the time series, indexing the degree of irregularity in local temporal structure of neural oscillations (Fig. 1).

### Neurodynamic profiles of Vipassana and Shoonya meditation: aims and hypotheses

This study aimed to investigate the neurodynamic profiles of Vipassana and Shoonya meditation by analyzing alpha oscillatory dynamics through PFS and PS. Beyond mean values, we assessed CV and PE on the PFS and PS timeseries to capture stability and temporal structure on the neural level in these two meditation styles.

Based on traditional descriptions and prior phenomenological reports (Hart 1987, Goenka 1995, Nash et al. 2013, Sadhguru 2016), we formulated operational hypotheses regarding the attentional-temporal styles associated with the two practices. Specifically, Vipassana was conceptualized as involving a comparatively detailed and sustained bodily focus, characterized by low variability and high temporal regularity. In contrast, Shoonya was conceptualized as less object-centered and less temporally structured, and thus as involving more variable and irregular attentional engagement. On this basis, we hypothesized that Vipassana would exhibit higher mean alpha PFS and PS, reflecting greater neural precision and intensity compared to Shoonya. Furthermore, given its sequential and structured deployment of attention, Vipassana was expected to show greater temporal stability and regularity of alpha dynamics, as indexed by lower variability (CV) and lower temporal irregularity (PE) in both PFS and PS. Conversely, Shoonya was predicted to exhibit higher temporal variability and greater temporal irregularity at the neural level, reflected in higher CV and PE of both alpha PFS and PS.

By comparing these two meditation techniques, this study seeks to clarify how alpha oscillatory dynamics differentiate between detailed versus non-specific, intense versus diffuse, stable versus variable, and regular versus irregular attentional styles promoted by two different meditation techniques. These findings may contribute to an empirical grounding of the theoretical proposal that neural activity and phenomenological experience share a common structure (i.e. “common currency” or “complex correspondence”; Northoff et al. 2020, Northoff and Lamme 2020, Northoff et al. 2024, Northoff and Ventura 2025), offering deeper insights into how temporal dynamics may serve as a bridge between neural activity and experience in different meditative states.

## Materials and methods

### Experimental setup

We performed the analyses on an open-access dataset that can be found at the following link: https://openneuro.org/datasets/ds003969/versions/1.0.0. The data were recorded using a 64 + 8 channels Biosemi Active-Two amplifier system and a 10–20 Headcap standard 64-channel cap from the same company, with an original sampling rate of 1024 Hz. External electrodes, including right and left mastoid electrodes, were utilized alongside a vertical and horizontal electrooculogram (EOG). For the EOG setup, two periocular electrodes were placed above and below the left eye, and one electrode at each of the left and right outer canthi. The procedure was conducted in a soundproof room with an electrically shielded and grounded floor. To ensure high-quality EEG signal, participants were instructed to wash their hair before the session. Additionally, the skin for non-scalp electrode placement was cleaned with an alcohol solution. All electrodes were maintained within a 50 mV offset according to the BIOSEMI system’s impedance measurement metric. For further details about the dataset (data collection, procedure, ethical approval, details about the meditation traditions, etcetera) the reader is referred to Braboszcz et al. (2017).

### Participants

The original dataset contained data from 20 Vipassana meditators (mean age = 47 ± 15), 27 Mantra meditators (mean age = 49 ± 13), 20 Shoonya meditators (mean age = 40 ± 10), and 32 meditation-naïve Controls (mean age = 45 ± 10), for a total of 98 participants. Here, we decided to include only Vipassana and Shoonya meditators to more effectively compare the influence of meditation on state features. In a previous publication using the same dataset (Ventura et al. 2024), these two groups were identified as representing the extremes of attentional focus, where Vipassana meditation was characterized by a narrow and sequential attentional focus on bodily sensations, and Shoonya meditation by a broad, unconstrained attentional scope. The groups’ estimated hours of lifetime meditation experience are summarized in Table 1. To ensure comparability between the two groups in terms of meditation proficiency, we conducted a Mann–Whitney U test on lifetime meditation hours. Two participants had missing data for this variable, resulting in a sample size of 32 for this analysis (Vipassana: n = 14; Shoonya: *n* = 18). The difference was not statistically significant, *U* = 173.5, *P* = .074, r = −0.38, 95% CI [−0.013, 0.667], indicating no meaningful difference in meditation experience between groups.

**Table 1:** Groups estimated hours of lifetime meditation experience.

Lifetime meditation hours	Vipassana meditators	Shoonya meditators
**Mean**	4 983	3 260
**SD**	3 616	2 866
**Minimum**	1 369	760
**Maximum**	12 319	10 403

### Data cleaning

Following automatic preprocessing (detailed in the Pre-processing section), the remaining sessions were manually reviewed, and those with evident artifacts were excluded. Approximately 3% of the data were missing, and as these were missing completely at random (unrelated to observed or unobserved data), Expectation Maximization was applied to impute values. This approach, an iterative statistical method for deriving maximum likelihood estimates with incomplete data, allowed us to achieve a final participant group that included 15 Vipassana meditators (mean age = 47 ± 16), and 19 Shoonya meditators (mean age = 38 ± 11), for a total of 34 participants. Furthermore, we winsorized the data at z = ±2.58, following statistical recommendations for our sample size.

### Ethics statement

Informed written consent before participation was obtained from all participants. The project was approved by the local MRI Indian ethical committee and the ethical committee of the University of California San Diego (IRB project #090731).

### Procedure

Participants practiced their regular meditation practices, as per the meditation tradition they belonged to. In particular, the Shoonya meditation group practiced Shoonya meditation, letting go of any specific attentional focus; and the Vipassana group practiced Vipassana meditation, focusing on a sequence of bodily stimuli. The sessions were performed with eyes closed.

### Pre-processing

EEG data preprocessing was conducted using the EEGLAB toolbox for MATLAB (R2023a; Delorme and Makeig 2004). To mitigate artifacts at the beginning and end of recordings, all 10-min sessions were trimmed to 9.5 min. The original sampling rate of 1024 Hz was down-sampled to 512 Hz using the resample function in EEGLAB. A band-pass filter ranging from 0.3 to 45 Hz was then applied to the continuous data. The clean_artifacts plugin in EEGLAB was utilized to address flatline channels, low-frequency drifts, noisy channels, and short-term bursts. Following this, the recordings were re-referenced to the average activity across all channels. Lastly, stationary artifacts, such as eye movements, breathing artifacts, and muscle-related noise, were corrected using independent component analysis and the MARA plugin in EEGLAB.

### Alpha Peak Sliding and Power Sliding analysis

We assessed the dynamics of alpha (7–13 Hz) peak frequency and power using the frequency sliding method developed by Cohen (2014). Below, we provide an overview of our procedure.

For each EEG channel, the preprocessed broadband EEG data were bandpass-filtered using a finite impulse response filter, with a 15% transition width. Next, the analytic signal was computed via the Hilbert transform, which allowed us to extract the phase angle time series. The phase angle at each time point is defined as the angle between the vector of the analytic signal and the real axis in the complex plane.

The instantaneous frequency was calculated as the first derivative of the phase angle time series. To mitigate noise effects, particularly those caused by abrupt “jumps” in the phase angle time series—common when estimating instantaneous frequencies in regions with low power (e.g. during unconscious states) — a median filter with a filter order of 100 was applied to the instantaneous frequency time series. At a sampling rate of 512 Hz, this corresponds to approximately 195 ms, well within the smoothing range recommended by Cohen (2014). Frequency sliding estimates are particularly susceptible to noise, and this temporal smoothing reduces noise-driven fluctuations while preserving physiologically meaningful alpha-band dynamics. Moreover, because PE is sensitive to noise and this may artificially inflate ordinal pattern complexity, stabilizing the instantaneous frequency signal was essential to ensure valid entropy estimation. The computation of the instantaneous power involved a similar strategy; however, instead of the phase angle time series, the squared magnitude, i.e. the modulus, of the analytic signal was considered.

Because our hypotheses concerned global attentional dynamics rather than spatially localized effects, PFS and PS time series were first computed separately for each channel and then averaged across all scalp electrodes, yielding a whole-cortex estimate of alpha dynamics for each participant. Accordingly, all reported metrics reflect spatially global effects.

### Permutation entropy

To investigate the temporal properties of the frequency (or, alternatively, power) in the alpha band during different meditative conditions, we applied permutation entropy (PE) (Bandt and Pompe 2002) on the frequency/power-sliding time series, which were described in the previous section. PE estimates the diversity of ordinal patterns in the observed time series (as many other Shannon Entropy-derived measures do), while taking into account temporal structure of information, therefore indexing temporal irregularity. The calculation of PE requires a symbolization procedure, which maps ordinal patterns into permutation patterns directly from the time series data. Below, we outline this procedure in detail (Riedl et al. 2013).

Given a timeseries $X=\left\{ Xt:t=1,\dots, N\right\}$, symbolization involves selecting two key parameters: the embedding dimension *D* and the embedding delay τ. These parameters govern the construction of embedding vectors, which we summarize step-by-step as follows:

1. For any chosen embedding dimension *D*, the time series is transformed into vectors of length *D*, consisting of temporally ordered values:
  $$Si=\left\{ Xi, Xi+\tau, Xi+2\tau, \dots, Xi+\left(D-1\right)\tau \kern0em \right\},i=1,2,\dots, N.$$

The embedding delay τ specifies the temporal gap to be considered between consecutive elements in each vector. For example, when τ =1, the original time granularity is preserved; when τ = 2, every other time point is used to construct the vectors.

2) Each vector $Si$ is transformed into an ordinal pattern by ranking its values in ascending order. The entries in $Si$ are replaced by their respective ranks, resulting in a sequence of ordinal patterns. Each ordinal pattern corresponds to a permutation pattern (a “symbol”), representing the relative order of the values in the embedding vector.
3) From the set of all possible permutation patterns, a probability distribution $P$is computed, where *p_i_* represents the frequency of the *i*th permutation pattern in the time series. For an embedding dimension *D*, there are $D!$possible permutations.
4) Finally, PE is defined as the Shannon entropy of the probability distribution *P*:
  $$PE=-\sum_{t=1}^{D!}{p}_i\ln{p}_i$$

where ${p}_i$​ is the probability of occurrence of the *i*th permutation pattern.

In this study, we parametrized the PE with an embedding delay τ = 1 and an embedding dimension *D* = 3. These parameters were selected following standard practice in EEG PE studies, where *D* = 3 and τ = 1 are commonly used to balance sensitivity to local temporal structure and statistical reliability (Bandt and Pompe 2002, Staniek and Lehnertz 2007). Given our sampling rate and focus on alpha-band dynamics, τ = 1 captures fine-grained temporal fluctuations within the alpha cycle, while *D* = 3 provides a robust estimate without overfitting shorter time windows. Robustness analyses using embedding dimensions *D* = 4 and *D* = 5 were conducted and are reported in the Supplementary material.

### Statistical analysis

Statistical analyses were conducted using JASP (JASP Team 2024), R (R Core Team, 2024), and RStudio (Posit team, 2024). Group comparisons were performed in JASP. Independent-samples *t*-tests were applied when parametric assumptions were satisfied, specifically when both the Shapiro–Wilk test of normality and the Brown–Forsythe test of equality of variances were non-significant, indicating no violation of normality or homoscedasticity assumptions. For measures that violated normality (Shapiro–Wilk *P* < .05), non-parametric Mann–Whitney *U* tests were used, as these do not assume normally distributed data. All tests were two-tailed with an alpha level of 0.05. For parametric tests, we report t values, degrees of freedom, two-tailed *P*-values, 95% confidence intervals, and Cohen’s *d* effect sizes; for non-parametric tests, we report U statistics and corresponding effect sizes and 95% confidence intervals. Effect sizes (Cohen’s *d*) reflect the group coding order (Shoonya − Vipassana).

To assess whether group differences in alpha-derived measures were influenced by potential confounding variables, additional regression analyses were conducted in R (see Supplementary material). For each outcome, ordinary least squares models were estimated with meditation group (Vipassana = 0; Shoonya = 1) as predictor, and models were progressively adjusted for age and for the log-transformed theta/alpha ratio (ratio between mean theta (4–7 Hz) and mean alpha (7–13 Hz) PS), included as an index of vigilance state. The ratio was log-transformed to reduce skewness. For PFS-derived measures, mean alpha PS was additionally included as a covariate to test whether effects on alpha PFS CV or PE were independent of oscillatory amplitude. Regression models employed heteroskedasticity-consistent (HC3) standard errors, and we report robust standard errors, t statistics, two-tailed *P*-values, 95% confidence intervals, *R*^2^, and sample size. Robust regression analyses were implemented using the *sandwich* and *lmtest* packages (Zeileis, 2004, 2006; Zeileis and Hothorn, 2002).

## Results

Statistical analyses of the independent samples were conducted to compare Shoonya and Vipassana meditation on the mean, CV, and PE values of PFS and PS in the alpha band. These metrics were derived from channel-wise PFS and PS time series that were averaged across all scalp electrodes, yielding a global estimate of alpha dynamics.

### Central tendency of alpha dynamics (mean)

In order to test for differences in the central tendency of alpha PFS and PS between Vipassana and Shoonya meditation, we calculated the mean values across timepoints in the metrics and we performed statistical tests.

**Figure 2:**
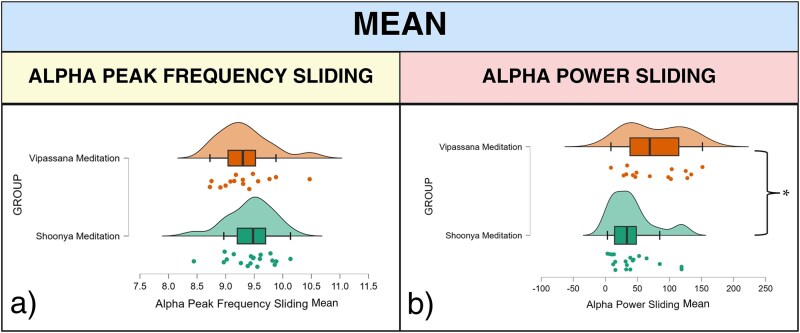
Differences in the mean values of alpha PFS and PS across Vipassana and Shoonya meditation practices. Panel (a) depicts the differences in mean PFS values and panel (b) illustrates the differences in mean PS values between the two meditation techniques. Significance levels indicated as: ^*^P ≤ .05.

Contrary to our hypothesis of higher alpha PFS in Vipassana compared to Shoonya meditation, an independent-samples *t-*test did not reveal a significant difference in mean PFS between Vipassana (*M* = 9.44, SD = 0.40) and Shoonya (*M* = 9.33, SD = 0.46), *t*(32) = −0.71, *P* = .486, *d* = −0.24, 95% CI [−0.44, 0.92] (Fig. 2a, Table 2).

**Table 2:** Group differences in alpha Peak Frequency Sliding (PFS) and Power Sliding (PS) mean, CV and PE between Vipassana and Shoonya meditation.

Outcome	Vipassana *M* (SD)	Shoonya *M* (SD)	Statistic (*t*)	df	*P*	Cohen’s *d*	95% CI
Mean							
Alpha PFS	9.44 (0.40)	9.33 (0.46)	−0.71	32	.486	−0.24	[−0.44, 0.92]
Alpha PS	76.96 (45.75)	40.96 (34.51)	−2.62	32	.013	−0.90	[−1.61, −0.19]
Coefficient of variation							
Alpha PFS	0.10 (0.014)	0.12 (0.02)	4.41	32	<.001	1.52	[0.74, 2.29]
Alpha PS	1.12 (0.14)	1.34 (0.19)	3.74	32	<.001	1.29	[0.54, 2.03]
Permutation entropy							
Alpha PFS	0.28 (0.014)	0.30 (0.02)	4.11	32	<.001	1.42	[0.65, 2.17]
Alpha PS	0.437 (0.003)	0.443 (0.005)	4.16	32	<.001	1.44	[0.66, 2.19]

Regarding mean alpha PS, an independent-samples *t-*test revealed that Vipassana meditation (*M* = 76.96, SD = 45.75) yielded significantly higher power than Shoonya meditation (*M* = 40.96, SD = 34.51), *t*(32) = −2.62, *P* = .013, *d* = −0.90, 95% CI [−1.61, −0.19] (Fig. 2b, Table 2). Regression models adjusting for age yielded consistent results, whereas adjustment for log-transformed theta/alpha ratio attenuated the PS mean effect to non-significance (see Supplementary Tables S1 and S2). Notably, Shoonya showed higher theta/alpha ratio than Vipassana, as reported in Supplementary material.

### Variability of alpha dynamics (coefficient of variation)

After testing for mean differences between Vipassana and Shoonya meditation in alpha PFS and PS, we wanted to assess changes in their variability. Therefore, we calculated the CV across timepoints and performed statistical tests.

**Figure 3:**
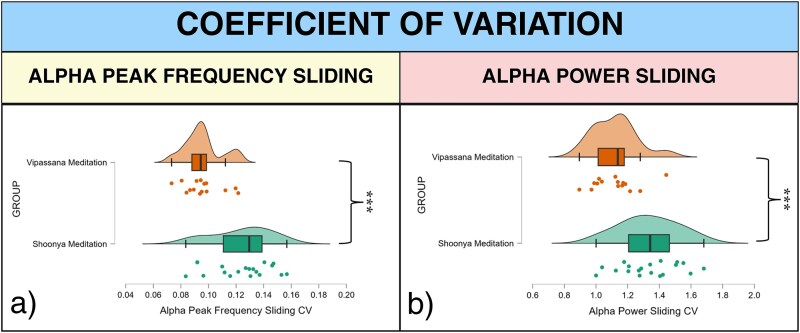
Differences in the coefficient of variation (CV) of alpha PFS and PS across Vipassana and Shoonya meditation practices. Panel (a) depicts the differences in CV of PFS values, and panel (b) illustrates the differences in CV of PS values between the two meditation techniques. Significance levels indicated as: ^***^P < .001.

Regarding the CV of alpha PFS, an independent-samples *t-*test revealed that the variability of the instantaneous peak was significantly lower in Vipassana meditation (*M* = 0.10, SD = 0.014) than in Shoonya meditation (*M* = 0.12, SD = 0.02), *t*(32) = 4.41, *P* < .001, *d* = 1.52, 95% CI [0.74, 2.29] (Fig. 3a, Table 2).

Similarly, regarding the CV of PS in the alpha band, an independent-samples *t-*test revealed that the variability of alpha power was significantly lower in Vipassana meditation (*M* = 1.12, SD = 0.14) than in Shoonya meditation (*M* = 1.34, SD = 0.19), *t*(32) = 3.74, *P* < .001, *d* = 1.29, 95% CI [0.54, 2.03] (Fig. 3b, Table 2). In regression models adjusting for age and log-transformed theta/alpha ratio, group differences remained statistically significant across outcomes (see Supplementary Tables S1 and S2). For PFS measures, effects also remained significant after additional adjustment for mean alpha PS (see Supplementary Table S1). In sum, Vipassana meditation showed lower variability in both alpha PFS and PS compared to Shoonya.

### Temporal irregularity of alpha dynamics (permutation entropy)

After addressing changes in the variability of alpha PFS and PS, we wanted to test for differences in temporal irregularity and structure. Therefore, we calculated the PE, indexing ordinal-pattern diversity, across timepoints and performed statistical tests.

**Figure 4:**
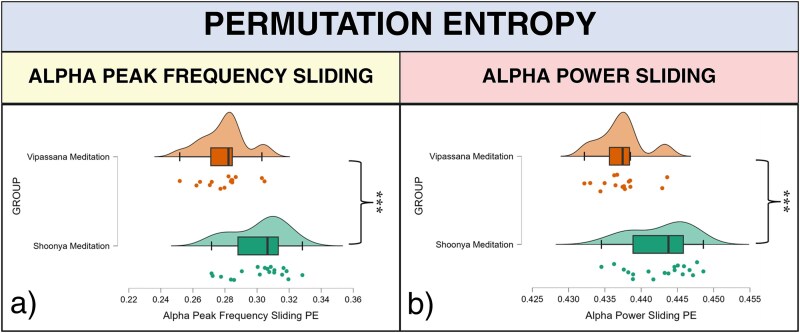
Differences in the permutation entropy (PE) of alpha PFS and PS across Vipassana and Shoonya meditation practices. Panel (a) depicts the differences in PE of PFS values, and panel (b) illustrates the differences PE of PS values between the two meditation techniques. Significance levels indicated as: ^***^P < .001.

Regarding the PE of alpha PFS, an independent-samples *t-*test revealed that the PE of the instantaneous peak was significantly lower in Vipassana meditation (*M* = 0.28, SD = 0.014) than in Shoonya meditation (*M* = 0.30, SD = 0.02), *t*(32) = 4.11, *P* < .001, *d* = 1.42, 95% CI [0.65, 2.17] (Fig. 4a, Table 2).

Similarly, regarding the PE of alpha PS, an independent-samples *t-*test revealed that the temporal irregularity of alpha power was significantly lower in Vipassana meditation (*M* = 0.437, SD = 0.003) than in Shoonya meditation (*M* = 0.443, SD = 0.004), *t*(32) = 4.16, *P* < .001, *d* = 1.44, 95% CI [0.66, 2.19] (Fig. 4b, Table 2). In regression models adjusting for age and log-transformed theta/alpha ratio, group differences remained statistically significant across outcomes (see Supplementary Tables S1 and S2). For PFS measures, effects also remained significant after additional adjustment for mean alpha PS (see Supplementary Table S1). Comparable group differences were observed across embedding dimensions (*D* = 3, 4, 5), indicating that the effects were consistent across parameter specifications (see Supplementary Figs S1–S4). In sum, Vipassana meditation exhibits lower PE in both alpha PFS and PS compared to Shoonya.

## Discussion

This study examined the neurodynamic profiles of Vipassana and Shoonya meditation by analyzing alpha oscillatory dynamics through PFS and PS. The results revealed significant differences in indices capturing the temporal organization of alpha dynamics (CV and PE), whereas differences in mean values were less consistent, with effects emerging for alpha PS but not for PFS. These findings tentatively suggest distinct patterns of neural dynamics in temporal resolution (PFS) and intensity (PS), which may correspond to differences in attentional style broadly consistent with theoretical descriptions of focused *versus* no-content meditation practices.

### Different temporal dynamics of alpha PFS in Vipassana and Shoonya meditation

The results indicate that the primary differentiation between Vipassana and Shoonya meditation in alpha PFS concerns its temporal dynamics rather than its central tendency. Although mean PFS did not differ significantly between groups, Vipassana showed markedly lower PFS variability (CV) and temporal irregularity (PE) compared to Shoonya.

First, the two practices differed in the stability (CV) of alpha PFS over time. Vipassana exhibited lower CV in alpha PFS than Shoonya, indicating that moment-to-moment fluctuations in oscillatory speed were more constrained and temporally stable. Shoonya, in contrast, exhibited higher CV, indicating greater moment-to-moment variability in oscillatory speed. Second, the two practices showed a similar pattern in the temporal regularity (PE) of alpha PFS fluctuations. As PE indexes the diversity of ordinal patterns in a time series, lower values correspond to more regular and structured temporal dynamics (Bandt and Pompe 2002, Staniek and Lehnertz 2007). Vipassana showed lower PE in alpha PFS than Shoonya, indicating greater temporal regularity in the organization of alpha frequency fluctuations, whereas Shoonya showed higher PE, reflecting more irregular temporal organization. Third, the absence of a significant group difference in mean alpha PFS suggests that the two practices do not differ in the average oscillatory speed of alpha activity. Rather, what distinguishes them appears to be how temporal resolution unfolds over time, i.e. its stability and structure. This dissociation may underscore the value of complementing mean-based characterizations with dynamic indices such as CV and PE, which could capture aspects of neural activity that static averages may neglect.

Because PFS is a phase-based measure reflecting the instantaneous “speed” of neural oscillations — and has been associated with temporal resolution in perceptual processing (Cohen 2014, Samaha and Postle 2015, Wutz et al. 2018, Benwell et al. 2019, Shen et al. 2019, Drewes et al. 2022, Jia et al. 2023) — the observed pattern of lower PFS variability (CV) and temporal irregularity (PE) may suggest that temporal resolution in the alpha band was both more stable and more structured across the meditation period in Vipassana, whereas higher PFS variability (CV) and entropy (PE) in Shoonya may reflect a more variable and more irregular temporal organization. Importantly, group differences in PFS CV and PE remained significant when statistically controlling for age, vigilance (log theta/alpha ratio), and mean alpha power (PS), suggesting that the observed effects are unlikely to be solely reducible to differences in arousal level or oscillatory amplitude alone (see Supplementary Table S1).

This dynamic contrast may align with traditional descriptions of Vipassana as involving a stable and sequential attentional deployment to different bodily regions, in which attention is systematically moved across the body according to a predefined structure (Hart 1987, Goenka 1995, Chiesa 2010, Delgado-Pastor et al. 2013, Nash et al. 2013, Braboszcz et al. 2017, Kakumanu et al. 2018). In such practice, attentional resolution may be progressively modulated within each bodily region—for instance, initially registering coarse sensations and then refining perceptual detail before shifting to the next region—resulting in a relatively stable and structured attentional pattern over time.

**Figure 5:**
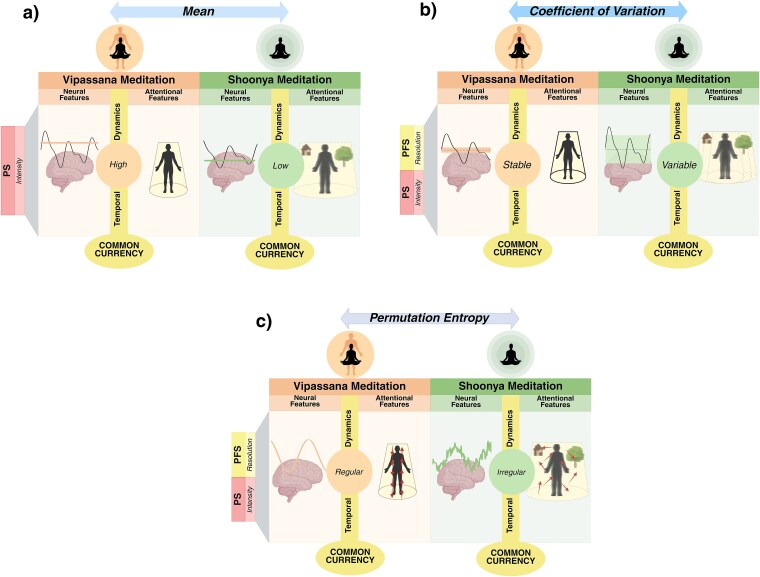
Neural and attentional profiles of Vipassana and Shoonya meditation. The two meditation styles can be positioned at opposite ends of continua (see arrows above each panel) in terms of PFS and PS dynamics. Within the present theoretical framework, PFS and PS are interpreted as candidate neural indices of temporal resolution and attentional intensity, respectively. The left side (orange) represents Vipassana, while the right side (green) represents Shoonya. Panel (a) illustrates group differences in the mean values of PS. Vipassana exhibited higher mean PS, whereas Shoonya showed lower mean PS. In the present operational framework, these differences may tentatively correspond to differences in the intensity of attentional engagement. Panel (b) illustrates differences in the coefficient of variation (CV) of PFS and PS. Lower CV in Vipassana indicates greater temporal stability of alpha dynamics, whereas higher CV in Shoonya reflects greater variability over time. Panel (c) illustrates differences in permutation entropy (PE) of PFS and PS. Lower PE in Vipassana indicates more temporally regular neural dynamics, whereas higher PE in Shoonya reflects greater temporal irregularity. Created in BioRender. Ventura, B. (2026); (a) https://BioRender.com/fyylbwy, (b) https://BioRender.com/fdplzzx, (c) https://BioRender.com/7uj9bbh.

In contrast, Shoonya has been described as involving a more fluid and free-floating attentional stance, open to whatever experience emerge without sequential engagement or deliberate modulation (Nash et al. 2013, Sadhguru 2016, Pagnoni 2019, Laukkonen and Slagter 2021). In this case, attentional resolution may fluctuate more variably and without a predefined structure, consistent with greater variability and temporal irregularity in neural dynamics (Fig. 5b and c). Crucially, these interpretations should be understood as theoretically grounded operational assumptions, rather than as established phenomenology directly measured in the present dataset.

### Different temporal dynamics of alpha PS in Vipassana and Shoonya meditation

The results indicate that Vipassana and Shoonya meditation differed in alpha PS at the level of both mean amplitude and its temporal dynamics.

First, at the level of mean alpha PS, Vipassana exhibited higher mean alpha amplitude than Shoonya. However, when statistically controlling for theta/alpha ratio, a well-known proxy of vigilance, the group difference in mean alpha PS was attenuated and no longer reached significance. This suggests that differences in mean alpha amplitude between the two practices may be partially related to arousal level.

Beyond central tendency, the two practices markedly differed in the temporal dynamics of alpha PS. Specifically, the two practices differed in the stability (CV) of alpha PS over time, with Vipassana exhibiting lower CV in alpha PS than Shoonya. This indicates that moment-to-moment fluctuations in oscillatory amplitude were more temporally stable. Shoonya, in contrast, showed higher CV, reflecting greater variability and reduced temporal stability in neural amplitude. A similar pattern emerged for the temporal structure (PE) of alpha PS fluctuations. Vipassana showed lower PE in alpha PS than Shoonya, indicating greater temporal regularity in amplitude fluctuations, whereas Shoonya showed higher alpha PS PE, reflecting more irregular temporal organization. Group differences in alpha PS CV and PE remained significant after controlling for age and theta/alpha ratio.

To interpret these differences, we situate alpha PS within the existing literature on oscillatory regulation and attentional control. Alpha PS reflects the instantaneous amplitude of the oscillatory signal, computed as the modulus of the Hilbert transform. Alpha amplitude has been associated with reduced cortical excitability and the gating of information between brain areas (Romei et al. 2008, Haegens et al. 2011, Lange et al. 2013, Benwell et al. 2019), as well as with attentional control and the filtering of irrelevant inputs (Klimesch et al. 2007, Jensen and Mazaheri 2010, Haegens et al. 2011, Van Diepen et al. 2019).

In this context, greater alpha amplitude has often been interpreted as reflecting a stronger engagement of top-down regulatory processes and a more selective allocation of neural resources. We adopted this literature-based framework to tentatively operationalize alpha PS as a possible neural correlate of attentional intensity, understood here not as a directly measured experiential variable but as the degree of sustained and selective engagement of neural processing with a given object or domain.

Taken together, these findings suggest that differences in mean alpha PS between the two meditation styles may partly depend on vigilance (i.e., theta/alpha ratio; see Supplementary Table S2). This pattern may indicate that the vigilance-related modulation of alpha PS might constitute a precondition for its possible role in attentional intensity. In other words, while alpha PS may be operationalized here as a neural correlate of attentional intensity, its expression appears to depend, at least in part, on the baseline level of arousal supporting sustained engagement. This interpretation is consistent with the observed group differences in theta/alpha ratio (see Supplementary material). In contrast, the temporal dynamics of alpha PS differ more robustly and consistently between the two practices, further highlighting the importance of complementing mean-based measurements with dynamic indices such as CV and PE.

These neural differences may align with traditional descriptions of Vipassana as involving sustained and deliberate engagement with bodily sensations during systematic body scanning (Hart 1987, Goenka 1995, Chiesa 2010, Delgado-Pastor et al. 2013, Nash et al. 2013, Braboszcz et al. 2017, Kakumanu et al. 2018), in which attentional intensity may be maintained and regulated in a more stable and structured manner.

In contrast, Shoonya has been described as involving relatively lower attentional intensity, with a more effortless and open awareness, without deliberate modulation of attention on specific objects (Nash et al. 2013, Sadhguru 2016, Pagnoni 2019, Laukkonen and Slagter 2021), which may be consistent with greater variability and less structured modulation of alpha neural amplitude observed in our results. As in the previous section, these interpretations should be understood as theoretically grounded operational assumptions rather than empirically-derived phenomenology from the present dataset.

Together, these findings indicate that Vipassana and Shoonya meditation differ primarily in the temporal dynamics of oscillatory alpha activity, both at the level of phase (PFS) and amplitude (PS), rather than in central tendency alone. Across both measures, the most consistent and pronounced distinctions concerned temporal stability (CV) and temporal structure (PE), suggesting that the two practices diverge more strongly in how neural dynamics unfold over time than in absolute levels of oscillatory frequency or power.

Importantly, the magnitude of effects varied across the neural metrics examined. While differences in mean alpha measures were generally small to moderate, indices capturing the temporal organization of neural dynamics, namely CV and PE of PFS and PS, showed large to very large effect sizes. This pattern reinforces the conclusion that Vipassana and Shoonya meditation are more robustly differentiated by the stability and regularity of their neurodynamic organization than by average oscillatory frequency or amplitude. In this sense, the primary contribution of the present study lies in showing that the brain’s neurodynamic indices of alpha PFS and PS reveal distinctions between structured and non-directed meditation practices which remain less visible when focusing on mean values alone.

### Methodological limitations and future directions

The present findings should be interpreted considering some methodological constraints. First, the sample size was modest (final *N* = 34), which limits statistical power and the generalizability of the findings. Large effect sizes should be interpreted cautiously given the modest sample size and may reflect sampling variability. Although consistent patterns emerged across multiple dynamic metrics, replication in larger cohorts will be necessary to confirm the robustness of these neurodynamic profiles. Second, the cross-sectional design prevents causal inference: while the observed differences in alpha dynamics are consistent with the distinct attentional styles associated with Vipassana and Shoonya meditation, we cannot determine whether these neural signatures result from long-term training, pre-existing individual differences, or their interaction. Third, the analyses relied on a secondary open-access dataset, which constrained control over recruitment procedures, inclusion/exclusion criteria, and potentially relevant contextual or practice-related variables that might differ between meditation traditions. These factors should be considered when interpreting the present findings.

Importantly, phenomenological measures were not collected in the present dataset; thus, interpretations linking neural dynamics to experiential features remain theoretically motivated operational assumptions rather than empirically demonstrated. In this view, integrating neurophenomenological paradigms could provide deeper insight into the link between neural oscillations and subjective experience. Combining EEG measures with first-person reports, such as micro-phenomenological interviews, questionnaires, and experience sampling, would allow researchers to map the dynamic interplay between neural activity and moment-to-moment shifts in awareness (Przyrembel and Singer 2018). This approach could clarify how stability and variability in neural signals correspond to phenomenological differences in attentional deployment, perceptual clarity, and self-experience across meditation styles. Another important direction for future studies is to incorporate an objective measure of external or internal input to assess how different meditation techniques modulate sensory or cognitive processing. This approach would provide a more direct way to evaluate how distinct meditation practices shape the temporal dynamics of input processing. By extending these findings across different meditation traditions, integrating neurophenomenological methodologies, and directly assessing input processing, future research can deepen our understanding of meditation’s impact on the brain and experience, further bridging contemplative science and neuroscience. Finally, given the modest sample size and the theoretical focus on global attentional dynamics, we did not perform topographic or region-specific analyses. Future work could examine whether these dynamic differences show spatial specificity across cortical regions.

## Conclusion

This study identified distinct neurodynamic signatures of alpha oscillations in Vipassana and Shoonya meditation. Using EEG, we showed that the two practices differed not only in mean alpha PS, but more prominently in the temporal organization of alpha dynamics. In particular, Vipassana exhibited greater temporal stability (lower CV) and regularity (lower PE) in both PFS and PS, whereas Shoonya showed greater variability (higher CV) and temporal irregularity (higher PE). Importantly, the most robust differences emerged in indices capturing the temporal dynamics of alpha PFS and PS, rather than in their mean levels alone. This highlights the critical role of neurodynamic measures, such as variability and entropy, in distinguishing structured from non-directed meditation practices.

More broadly, these findings contribute to the growing body of research on meditation and the brain’s neurodynamics by highlighting the role of temporal metrics in characterizing attentional modes at the neural level. In line with recent proposals on the shared temporal features of brain and mind (Northoff et al. 2020, Northoff and Ventura 2025, Northoff and Lamme 2020), our results support the view that temporal stability and structure may constitute a “common currency” linking neural dynamics and experiential organization in distinct ways in different contemplative practices.

## Supplementary Material

Supplementary_AlphaDynamics_BV_niag036

## Data Availability

The data underlying this article are publicly available in OpenNeuro at https://openneuro.org/datasets/ds003969/versions/1.0.0.
