# Supplementary material for: Neurodynamic profiles in the alpha band distinguish different forms of meditation
Source: Neurosci Conscious. 2026 Jul 7;2026(1):niag036. doi: 10.1093/nc/niag036 (PMC13338902; doi:10.1093/nc/niag036)
Supplement: Supplementary_AlphaDynamics_BV_niag036 [file supplementary_alphadynamics_bv_niag036.docx]

**Supplementary Materials**

**Table S1**

Robustness Regressions (HC3): Group Effects (Shoonya vs. Vipassana) on Alpha Frequency Peak Sliding (PFS) Outcomes

| Outcome | Model | B | SE (HC3) | t | p | 95% CI [LL, UL] | R² |
| --- | --- | --- | --- | --- | --- | --- | --- |
| Alpha PFS Mean | Group | 0.105 | 0.156 | 0.67 | .507 | [−0.212, 0.422] | .015 |
|  | Group + Age | 0.053 | 0.189 | 0.28 | .781 | [−0.333, 0.439] | .046 |
|  | Group + Vigilance (log θ/α) | 0.226 | 0.147 | 1.54 | .135 | [−0.074, 0.525] | .147 |
|  | Group + Alpha power (PS mean) | −0.019 | 0.147 | −0.13 | .896 | [−0.319, 0.280] | .116 |
| Alpha PFS CV | Group | 0.028 | 0.006 | 4.51 | < .001 | [0.016, 0.041] | .378 |
|  | Group + Age | 0.031 | 0.007 | 4.52 | < .001 | [0.017, 0.045] | .399 |
|  | Group + Vigilance (log θ/α) | 0.019 | 0.006 | 3.34 | .002 | [0.007, 0.031] | .646 |
|  | Group + Alpha power (PS mean) | 0.018 | 0.006 | 3.15 | .004 | [0.006, 0.029] | .627 |
| Alpha PFS PE | Group | 0.022 | 0.005 | 4.08 | < .001 | [0.011, 0.033] | .346 |
|  | Group + Age | 0.024 | 0.006 | 4.12 | < .001 | [0.012, 0.035] | .359 |
|  | Group + Vigilance (log θ/α) | 0.013 | 0.004 | 3.11 | .004 | [0.004, 0.021] | .747 |
|  | Group + Alpha power (PS mean) | 0.014 | 0.005 | 2.67 | .012 | [0.003, 0.025] | .554 |

Note. Each row reports the estimated effect of Group (coded 1 = Shoonya, 0 = Vipassana) from a separate linear regression model with HC3 robust standard errors. Robustness regressions were estimated using heteroskedasticity-consistent standard errors (HC3) to account for potential violations of homoscedasticity, which is recommended for small samples.

**Table S2**

Robustness Regressions (HC3): Group Effects (Shoonya vs. Vipassana) on Alpha Power Sliding (PS) Outcomes

| Outcome | Model | B | SE (HC3) | t | p | 95% CI [LL, UL] | R² |
| --- | --- | --- | --- | --- | --- | --- | --- |
| Alpha PS Mean | Group | −36.000 | 14.700 | −2.45 | .020 | [−65.900, −6.090] | .176 |
|  | Group + Age | −39.500 | 16.100 | −2.46 | .020 | [−72.300, −6.770] | .190 |
|  | Group + log θ/α | −30.100 | 16.100 | −1.88 | .070 | [−62.900, 2.630] | .207 |
| Alpha PS CV | Group | 0.216 | 0.058 | 3.76 | < .001 | [0.099, 0.333] | .304 |
|  | Group + Age | 0.228 | 0.067 | 3.41 | .002 | [0.092, 0.365] | .312 |
|  | Group + log θ/α | 0.180 | 0.065 | 2.78 | .009 | [0.048, 0.313] | .358 |
| Alpha PS PE | Group | 0.006 | 0.002 | 4.18 | < .001 | [0.003, 0.009] | .350 |
|  | Group + Age | 0.007 | 0.002 | 4.26 | < .001 | [0.004, 0.010] | .367 |
|  | Group + log θ/α | 0.004 | 0.001 | 3.05 | .005 | [0.001, 0.006] | .720 |

Note. Each row reports the estimated effect of Group (coded 1 = Shoonya, 0 = Vipassana) from a separate linear regression model with HC3 robust standard errors. Robustness regressions were estimated using heteroskedasticity-consistent standard errors (HC3) to account for potential violations of homoscedasticity, which is recommended for small samples.

**Theta/Alpha PS Ratio**

Theta/Alpha PS ratio differed significantly between Shoonya and Vipassana according to a Mann–Whitney test, U = 204, p = .033, rank-biserial r = −0.43, 95% CI [−0.70, −0.07], with Shoonya (M = 0.571, SD = 0.264) showing higher values than Vipassana (M = 0.397, SD = 0.179).


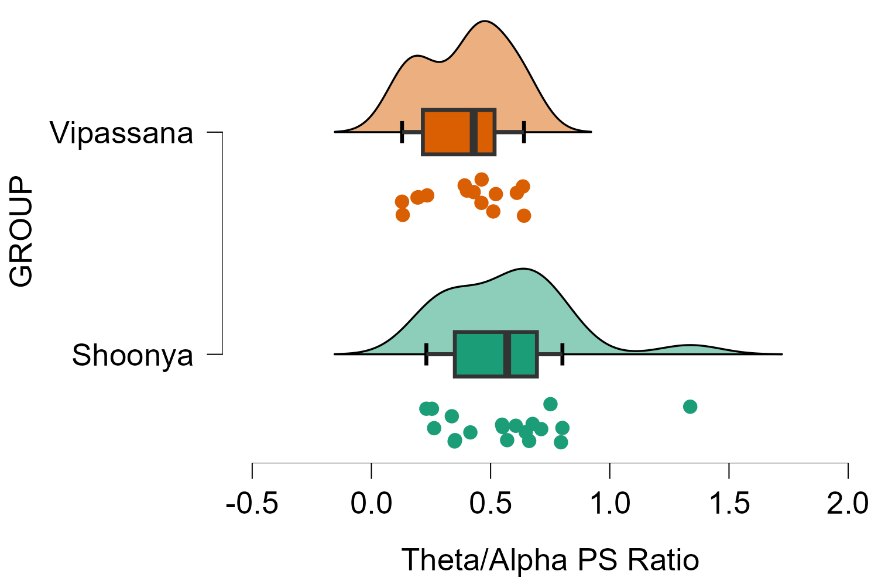


**Permutation Entropy Embedding Dimension Robustness Analyses**

To examine whether the observed group differences in permutation entropy (PE) depend on the embedding dimension, PE was computed using D = 4 and D = 5 (τ = 1), in addition to the original D = 3 analysis (see main manuscript.

Assumption checks confirmed normality (all Shapiro–Wilk p ≥ .152) and homogeneity of variances (all Brown–Forsythe p ≥ .129) across all models.

**Alpha Peak Frequency Sliding (PFS) PE**

**D = 4**

Shoonya practitioners (n = 19, M = 0.228, SD = 0.013) showed significantly higher PE values than Vipassana practitioners (n = 15, M = 0.211, SD = 0.011), t(32) = 4.18, p < .001, Cohen’s d = 1.44, 95% CI [0.67, 2.20] (Supplementary Figure 1).


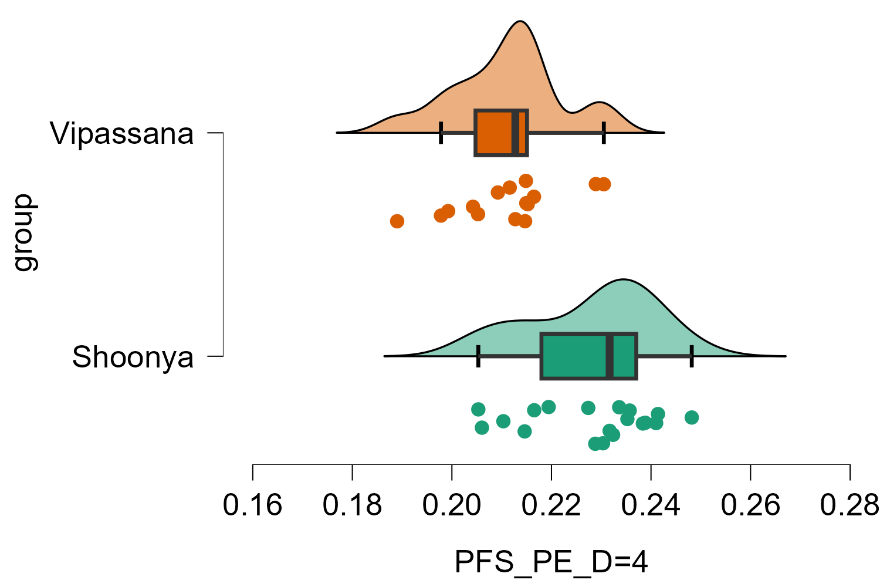


*Supplementary Figure 1: Alpha PFS PE (PFS_PE, D = 4) during Vipassana vs Shoonya meditation.*

**D = 5**

The group difference remained significant. Shoonya (M = 0.188, SD = 0.010) showed higher PE than Vipassana (M = 0.174, SD = 0.009), t(32) = 4.23, p < .001, Cohen’s d = 1.46, 95% CI [0.69, 2.22] (Supplementary Figure 2).


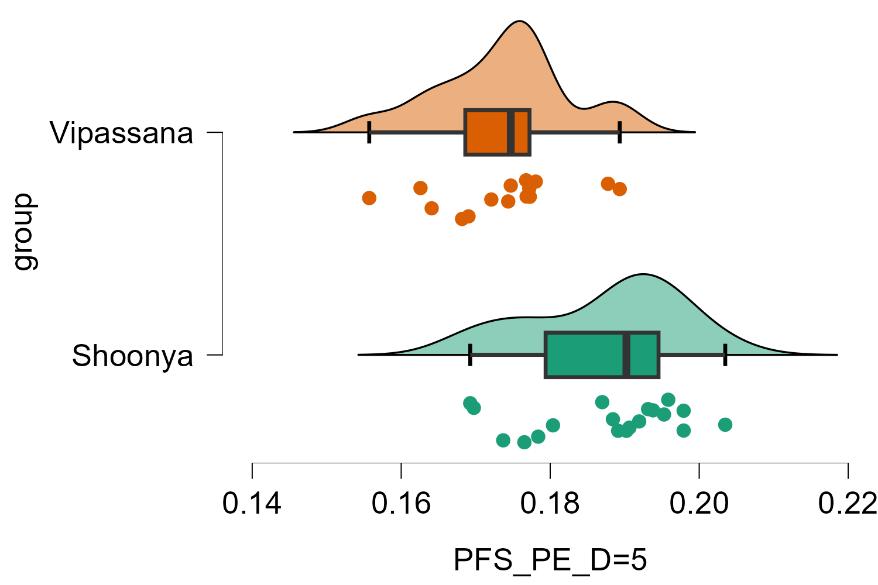


*Supplementary Figure 2:* *Alpha PFS PE (PFS_PE, D = 5) during Vipassana vs Shoonya meditation.*

**Alpha Power Sliding (PS) PE**

**D = 4**

Shoonya (M = 0.282, SD = 0.005) showed higher PE than Vipassana (M = 0.276, SD = 0.004), t(32) = 4.17, p < .001, Cohen’s d = 1.44, 95% CI [0.67, 2.20] (Supplementary Figure 3).


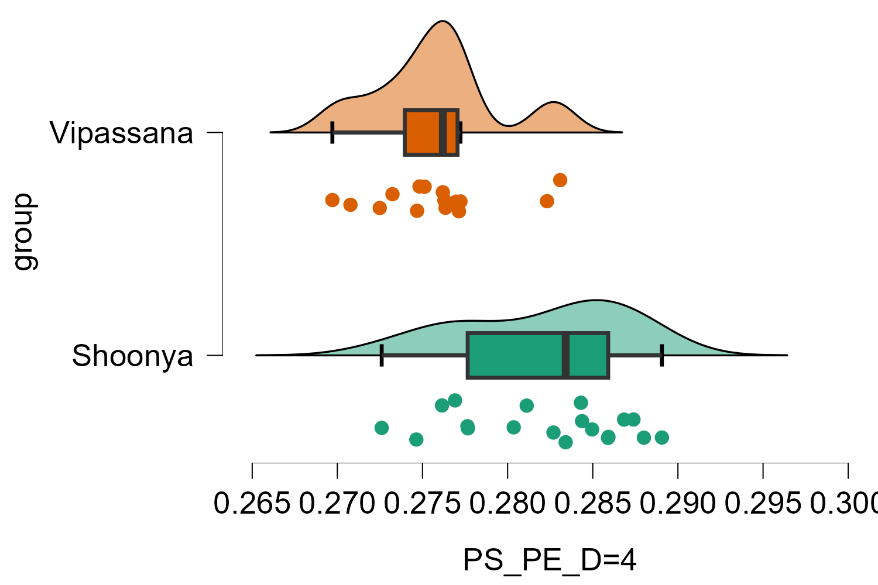


*Supplementary Figure 3: Alpha PS PE (PS_PE, D = 4) during Vipassana vs Shoonya meditation.*

**D = 5**

The group difference remained robust. Shoonya (M = 0.209, SD = 0.005) vs Vipassana (M = 0.202, SD = 0.004), t(32) = 4.18, p < .001, Cohen’s d = 1.44, 95% CI [0.67, 2.20] (Supplementary Figure 4).


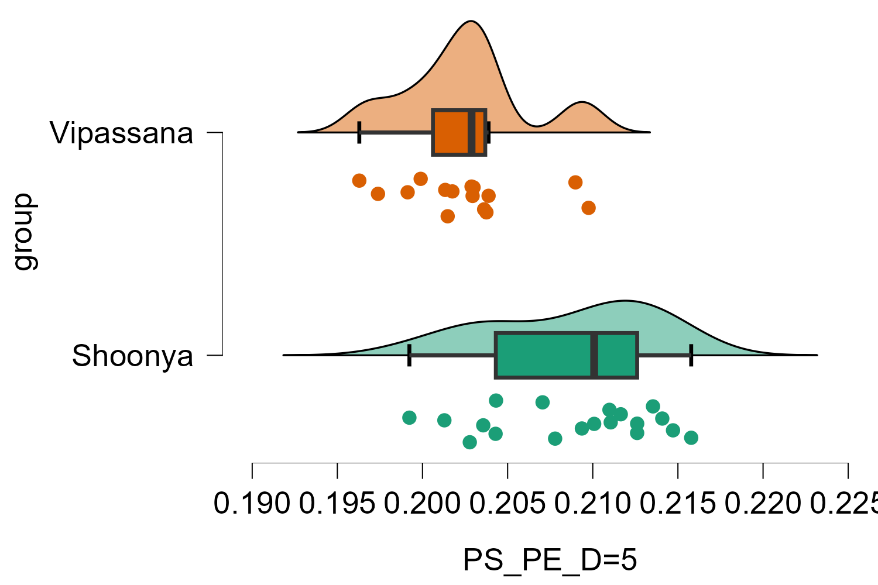


*Supplementary Figure 4:* *Alpha PS PE (PS_PE, D = 5) during Vipassana vs Shoonya meditation.*

Across embedding dimensions (D = 3, 4, 5), Shoonya practitioners consistently showed higher alpha PFS and PS PE than Vipassana practitioners. The effect remained significant with large and stable effect sizes (Cohen’s d ≈ 1.44–1.46).
